# Direct atomic insight into the role of dopants in phase-change materials

**DOI:** 10.1038/s41467-019-11506-0

**Published:** 2019-08-06

**Authors:** Min Zhu, Wenxiong Song, Philipp M. Konze, Tao Li, Baptiste Gault, Xin Chen, Jiabin Shen, Shilong Lv, Zhitang Song, Matthias Wuttig, Richard Dronskowski

**Affiliations:** 1 State Key Laboratory of Functional Materials for Informatics, Shanghai Institute of Micro-System and Information Technology, Chinese Academy of Sciences, 200050 Shanghai, China; 2 Chair of Solid-State and Quantum Chemistry, Institute of Inorganic Chemistry, RWTH Aachen University, 52056 Aachen, Germany; 3 Max-Planck-Institut für Eisenforschung GmbH, 40237 Düsseldorf, Germany; 4 Department of materials, Imperial College London, London, SW7 2AZ UK; 5 I. Institute of Physics (IA), RWTH Aachen University, 52056 Aachen, Germany; 6 Peter Grünberg Institute (PGI 10), Forschungszentrum Jülich, 52428 Jülich, Germany; 7 Jülich-Aachen Research Alliance (JARA-HPC), RWTH Aachen University, 52056 Aachen, Germany; 8 Hoffmann Institute of Advanced Materials, Shenzhen Polytechnic, 7098 Liuxian Blvd, Nanshan District, Shenzhen, China

**Keywords:** Information storage, Phase transitions and critical phenomena

## Abstract

Doping is indispensable to tailor phase-change materials (PCM) in optical and electronic data storage. Very few experimental studies, however, have provided quantitative information on the distribution of dopants on the atomic-scale. Here, we present atom-resolved images of Ag and In dopants in Sb_2_Te-based (AIST) PCM using electron microscopy and atom-probe tomography. Combing these with DFT calculations and chemical-bonding analysis, we unambiguously determine the dopants’ role upon recrystallization. Composition profiles corroborate the substitution of Sb by In and Ag, and the segregation of excessive Ag into grain boundaries. While In is bonded covalently to neighboring Te, Ag binds ionically. Moreover, In doping accelerates the crystallization and hence operation while Ag doping limits the random diffusion of In atoms and enhances the thermal stability of the amorphous phase.

## Abstract (web summary)

Quantitative imaging on the doping in phase-change materials for data storage remains scarce. Here, the authors combine electron microscopy, atom probe tomography, and simulations to determine the role of indium and silver dopants during recrystallization.

## Introduction

Over the last 50 years, computers have revolutionized almost every aspect of modern life, in particular communication, education, entertainment, and science, too. Today, however, they face increasing demands for faster data access and larger storage capacity, which are both severely limited by the presently available memory (fast, volatile, small) and storage (slow, non-volatile, large) hierarchies^1,2^. One successful approach for technological improvement is the introduction of phase-change memory^3^, also marketed by Intel/Micron as 3D Xpoint^4^. It takes advantage of the ability to switch the resistance upon the phase transition from a disordered amorphous (logic 1) to an ordered crystalline phase (logic 0) in certain chalcogenides, providing nonvolatility, nanoseconds speed and 4F^2^ (F: feature size) density^5,6^, thereby bridging the performance gap between memory and storage^3^. The successful commercialization of phase-change memory has been enabled by successful doping chalcogenides in the pseudo-binary GeTe–Sb_2_Te_3_ family^7,8^, as well as Ag and In doping Sb_2_Te alloy abbreviated as AIST, in which between 3.4 at. % and 11 at. % Ag and In have been incorporated^9,10^. Thanks to enhanced erasability and sensitivity, AIST has been frequently used in rewritable optical-storage media such as CD-RW and DVD-RW, already since 1993^9,10^. Moreover, AIST is capable of recrystallizing on the nanosecond time scale from an amorphous-crystalline rim. This enables growth-dominated recrystallization behavior^11,12^, which offers increased potential for DRAM/SRAM-like phase-change memory applications.

Until now, however, very few experimental studies have provided quantitative information on the role of the Ag and In dopants in AIST, due to limits of analytical technology. This hinders further material optimizations to boost device performance, especially with regards to operation speed and device lifetime. In the earliest report from 1993, Iwasaki et al. employed X-ray diffraction (XRD) and transmission electron microscopy (TEM) and showed that AIST, with 11 at.% Ag and In, crystallized not as a single phase but as a solid mixture of AgInTe_2_ and Sb phases after annealing at 230 °C^10^. They also observed a solid-state reaction of these phases to yield AgSbTe_2_ and InSb as the temperature increases to 350 °C. In 2001, Matsunaga et al. utilized synchrotron-radiation XRD and proposed a uniform A7 crystal structure (*R*$\bar 3$*m*) with just one crystallographic site, randomly occupied by all atoms (Ag, In, Sb, or Te). It subsequently changes upon annealing to a rhombohedral structure at ~507 °C^13^. In contrast, Raoux et al. discovered that the XRD pattern of AIST resembled pure Sb_2_Te, characterized by the repeated stacking of Sb_2_ bilayers and Sb_2_Te_3_ quintuple layers^14^. Based on a statistical technique called fluctuation TEM, Lee et al. directly detected the nanometer-scale nuclei embedded in AIST and their evolution over time^15^. Later, Matsunaga et al. combined extended X-ray absorption fine structure (EXAFS), hard X-ray photoelectron spectroscopy (HXPS) and reverse Monte Carlo (RMC) simulations, and determined a distortedly octahedral atomic arrangement of Sb and Te atoms but were less successful to explain the precise role of Ag and In atoms^12^. In both aforementioned cases, as well as in the recent density-functional theory (DFT) calculations^16^, a randomly mixed structure of AIST was used.

In this contribution, we provide atomic insight of the distribution of 5.13 at.% Ag and 3.32 at.% In within polycrystalline thin films of AIST, the composition of which is close to that used in rewriteable optical-storage media such as DVD-RW (~5 at.% Ag and ~5 at.% In)^5^. First their occupancy in the main structure of the grains is revealed by atomically resolved aberration-corrected scanning TEM (Cs-corrected STEM). In addition, quantitative information is provided by atom-probe tomography (APT) performed specifically at grain boundaries. Ultimately, the structure model obtained is fed into density-functional theory calculations and analyzed by chemical-bonding theory, through which the precise role of each element in the recrystallization process is revealed.

## Results

### Structure and chemical identifications

**Fig. 1:**
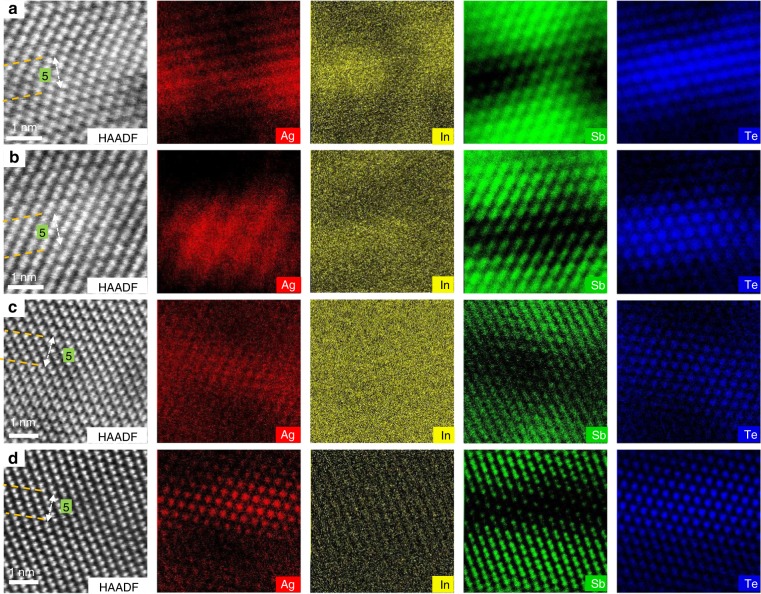
Atomic structure of crystalline AIST annealed at different temperatures. **a**‒**d** HAADF-STEM images, and corresponding EDX mappings of Ag, In, Sb and Te elements annealed at 200, 250, 300, and 350 °C, respectively. The scale bar is 1 nm

Figure 1 presents the atomic arrangement and element distributions of crystalline AIST obtained from in situ TEM investigation. The atomically resolved high-angle annular dark-field (HAADF) images in Fig. 1a show that crystalline AIST annealed at 200 °C is stacked by both bilayer and quintuple-layer units. Ag, In, Sb, and Te are distributed inhomogeneously, with Ag and In showing a preference to the Sb_2_Te_3_ quintuple layers, while the bilayers are built from mostly Sb. After increasing the temperature to 350 °C (Fig. 1b–d), the changes in atomic arrangement are miniscule, while the degree of crystallinity increases with higher heating temperature, as indicated by clearer atomic energy-dispersive X-ray spectroscopy (EDX) mappings.

**Fig. 2:**
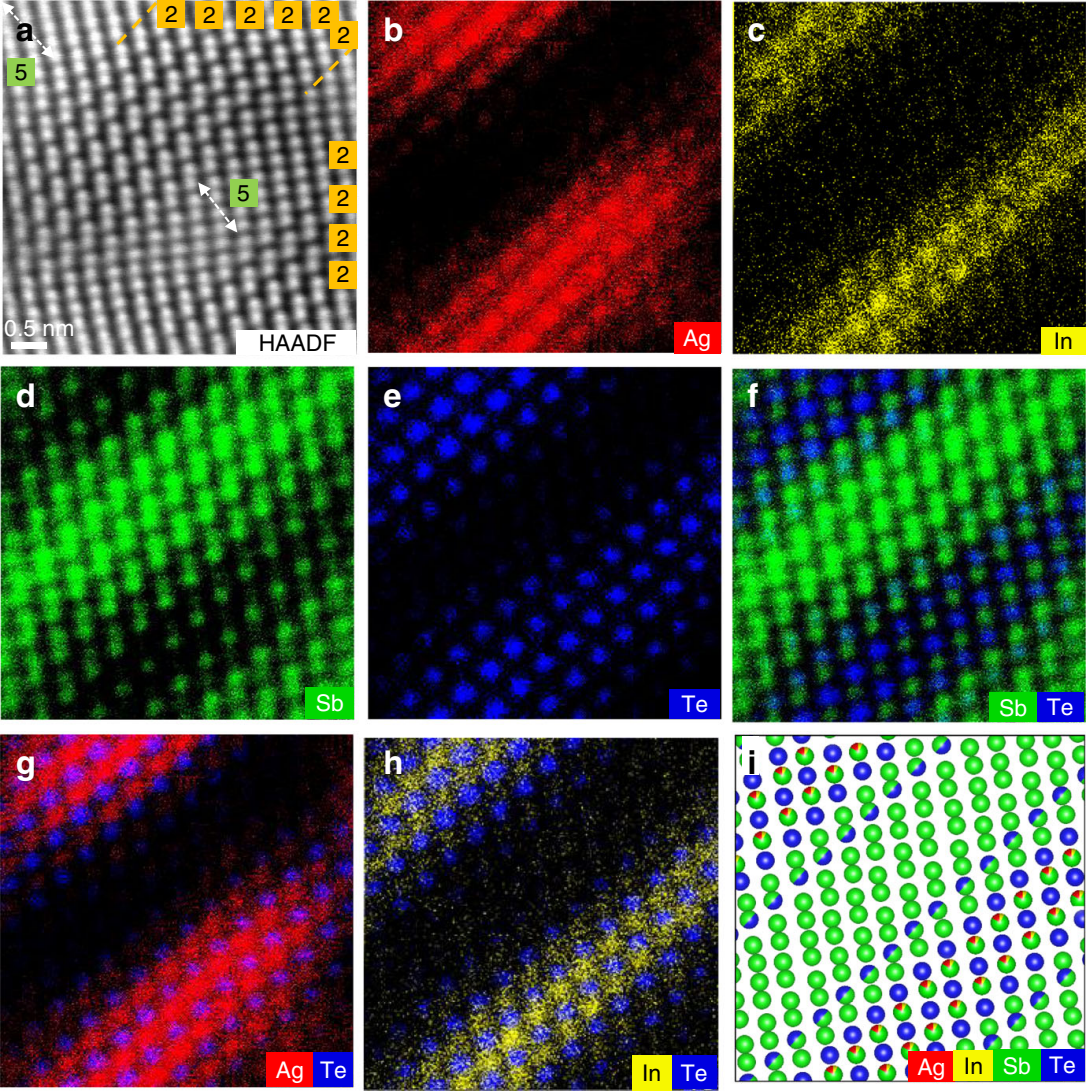
Structural and chemical identifications of an AIST crystallite, stacked by bilayers and quintuple layers. **a** HAADF-STEM image; the arrows mark the quintuple layers between two bilayers. **b**–**h** EDX mappings for Ag, In, Sb, Te, Sb + Te, Ag + Te, and In + Te elements, respectively. I: corresponding schematic atomic stacking model of crystalline AIST. The scale bar is 0.5 nm

To investigate the influence of annealing time on the atomic arrangement, the sample was annealed at 300 °C for 30 min, so Fig. 2 shows additional micrographs after this annealing step. Apparently, as seen from the HAADF image in Fig. 2a, crystalline AIST shows a bilayer stacking sequence, separated by narrow gaps, which is commonly found in crystalline AIST^13^. The observed atomic arrangement remains the same as that observed in Fig. 1, which suggests that the crystalline structure of AIST is barely affected by the annealing time. Correspondingly, the line profile taken across four bilayers shows two kinds of atomic column distances (Supplementary Fig. 1), 2.06 Å and 2.61 Å, in line with the Matsunaga model (Supplementary Fig. 2)^13^. The atomic configuration is identical to that of the rhombohedral (A7-type) Sb crystal projected along the <001> direction^17^. A ~20% variation in bond lengths (three shorter and three longer bonds) is observed, which is attributed to the three-dimensional energy-lowering Peierls distortion of the ideal rocksalt structure^18^. These bilayer structures (marked by yellow boxes) are replaced in some regions, as shown in Fig. 2a, by quintuple layer (highlighted by green boxes). Two interplanar distances are shown in the quintuple layer (Supplementary Fig. 1F), namely 2.22 Å and 2.34 Å, slightly larger than those of the interlayer distances. The quintuple layer is surrounded by bilayers at a spatial width of 2.48 Å. Thereby, AIST has a crystalline structure resembling undoped Sb_2_Te, characterized by a systematic stacking of ordered bi- and quintuple-layer blocks (as proposed by Kifune et al.^19^), which is significantly different from the repeated bilayer stacking in the Matsunaga model^13^. However, these two building blocks of Sb_2_Te are randomly distributed in crystalline AIST (comparing Fig. 2 and Supplementary Fig. 1), lacking the distinct order found in the binary compound.

While the intensity of the atoms in HAADF images is approximately proportional to *Z*^1.7^ (ref. ^20^), suggesting a Te-Sb-Te-Sb-Te quintuple layer, the figures still lack information regarding Ag and In. This is attributed to the presence of four elements with similar atomic numbers and low Ag and In concentrations. Further atom-resolved EDX was used to resolve the occupancy, as shown in Fig. 2b to h. Apparently, as seen from Fig. 2a, two quintuple blocks are separated by five continuously stacked bilayers. Indeed, the stacking sequence of a quintuple layer is Te1-Sb-Te2-Sb-Te1, as mentioned above, which can be observed directly from the Sb, Te and their overlay mappings in Fig. 2d to f, respectively. Noticeably, as shown in Fig. 2b, c, the atomically resolved signals from Ag and In atoms stem from the same sites within the quintuple layers as the Sb atoms. These dopants thus substitute Sb, and are hence dubbed Ag_Sb_ and In_Sb_ with the Sb subscript denoting the substitution site. They are more visualized in the overlaid Ag + Te and In + Te in Fig. 2g, h, respectively. In contrast, Sb exclusively occupies the bilayers between the Te-Sb-Te-Sb-Te quintuple layers, and no significant amount of Te, Ag, or In can be detected inside the bilayers. As shown in Supplementary Fig. 3, Ag_Sb_ and In_Sb_ defects are dramatically reduced as a defective quintuple-layer splits into bilayer and triple-layer (with a small gap between them), as also observed in the crystalline Ti-Sb-Te alloy^21,22^. Summarizing, the atomic stacking model of crystalline AIST is schematically presented in Fig. 2i. The layered stacking configuration obviously differs from the randomly mixed model proposed by Matsunaga et al. in 2001^13^.

### Element distributions in the grain boundary

**Fig. 3:**
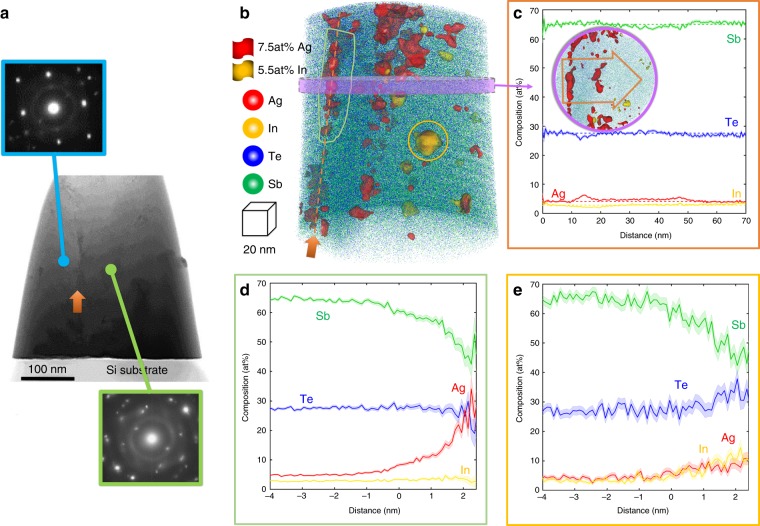
Correlative APT-TEM investigation of grain boundary in crystalline AIST. **a** HAADF-STEM image of the needle-shaped AIST specimen along with the diffraction patterns from selected areas evidencing the high-angle grain boundary. **b** APT mapping of the AIST showing the segregation of Ag. **c** concentration profiles and ion count of the region of interest highlighted by blue cylinder. Proximity histogram concentration profiles of Ag, In, Sb, and Te for D: 7.5 at.% Ag and E: 5.5 at.% In iso-concentration surfaces. APT results show that ~3.9 at.% Ag and ~3.3 at.% In substitute the Sb in the quintuple layer, and excessive Ag segregates into the grain boundary

Noticeably, Ag atoms not only locate in the Sb_2_Te_3_ quintuple layer, forming Ag_Sb_ defects, but also aggregate in the grain boundary (GB), as shown in the EDX mappings in Supplementary Fig. 4. In contrast, fewer In, Sb and Te atoms are found in the grain boundary, supported by the lower intensity than those inside the grains. To provide quantitative information on the distribution of Ag within both grains and GB, we performed correlative TEM-atom-probe tomography investigations. APT has high elemental sensitivity (>1‰) and provides sub-nanometer-scale mapping in three-dimensions^23^. The principle of APT is illustrated in the method section and highlighted in ref. ^24^. Figure 3A shows the HAADF-STEM image of the needle-shaped APT specimen. The presence of a high-angle GB is assessed by the different diffraction patterns on either side of the feature highlighted by the red arrow. Figure 3B presents the corresponding reconstructed three-dimensional (3D) mapping obtained from APT. The location of the grain boundary is indicated by the dark orange dashed arrow. A set of iso-surfaces encompassing regions of the point cloud containing over 7.5 at.% Ag and 5.5 at.% In are overlaid onto the point cloud in red and yellow, respectively. The thresholds were adjusted to highlight the non-homogeneous distribution of these elements throughout the microstructure. The one-dimensional composition profile displayed in Fig. 3c was calculated along a *d* = 50 nm cylindrical region-of-interest positioned normal to the grain boundary, as indicated in the inset, in which a top-view of the grain boundary is displayed. The dashed lines correspond to the average composition within the grains. Inside both grains, the composition is 65.5 at.% Sb, 26.6 at.% Te, 3.9 at.% Ag, and 3.3 at.% In. The grain boundary is In-depleted, with a composition dropping to ~2 at.%, which contrasts with a statistically significant segregation of Ag up to 6 at.%. This result is consistent with the EDX mappings in Supplementary Fig. 4. From the grain boundary, a 40 nm-thick region extends into the grain. This layer is slightly enriched in Ag, to ~5 at.%, and in Te at 27 at.%, accompanied by a corresponding depletion of Sb to 63.5 at.% and of In to 2.8 at.%. This means that all In atoms substitute Sb atoms, whereas excessive Ag appears segregated at the grain boundary.

An average composition profile for 14 Ag-rich particles sitting at the boundary was calculated as a function of the distance to the isosurface (referred to as a proximity histogram^25^) and is shown in Fig. 3d. The Ag composition can reach up to over 33 at.%, indicative of a strong tendency for phase separation. An Ag particle is also directly observed in a low-angle GB in Supplementary Fig. 5, the core of which is indeed free of In/Sb/Te. In addition, In-rich clusters in the range of 5–10 nm in diameter are observed inside the grains but not at the GB or the interface. The composition profile calculated as a function of the distance to the In-isosurface, shown in Fig. 3e, evidences the coexistence of In and Ag, accompanied by a strong depletion in Sb and a slight increase in Te. This suggests that these areas are akin to the quintuple layer with Ag_Sb_ and In_Sb_, as found in the EDX mapping in Fig. 2.

### Crystal orbital Hamilton population and chemical-bonding analysis

**Fig. 4:**
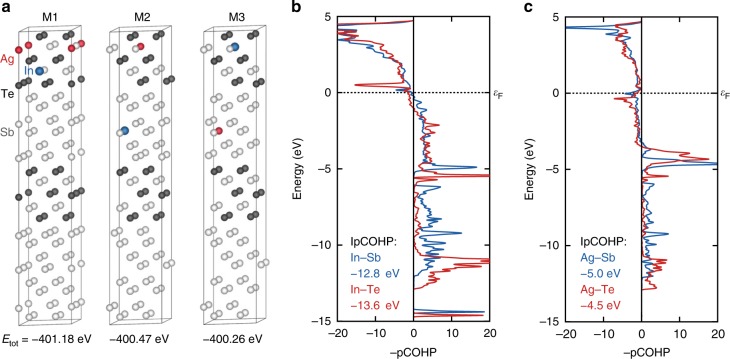
Total energy and bonding analysis of AIST with different arrangements. **a** structural images of three typical AIST models (M1–3) including their total electronic energy. **b** projected COHP (pCOHP) plots of In–Te (red) and In–Sb (blue) interactions in models M1 and M2, respectively. **c** pCOHP plots of Ag–Te (red) and Ag–Sb (blue) interactions in models M1 and M3, respectively. The integral of the pCOHP up to the Fermi level (IpCOHP) is shown to better quantify the results. The energy axes are referenced to the Fermi level ε_F_

Through Cs-TEM and APT, we qualitatively determine the chemical and structural arrangement of crystalline AIST as follows: Sb and Te form Te-Sb-Te-Sb-Te quintuple layers, which are separated by randomly stacked Sb-Sb bilayers; ~3.9 at.% Ag and ~3.3 at.% In substitute the Sb in the quintuple layer, forming Ag_Sb_ and In_Sb_ point defects, whereas excess Ag (above 3.9 at.%) separates in or along GBs. Hence, both Cs-corrected and APT results suggest consistently that there are basic units in AIST with different atomic arrangements, different composition, as well as different dopant distribution. These results also explain the phase segregation in AIST with 8–11 at.% Ag and In, as observed by Iwasaki et al.^10^, while a single rhombohedral phase was found in AIST with only 3.4 at.% Ag and 3.7 at.% In by Matsunaga et al.^13^. No In segregation is found because the maximum solubility of In_Sb_ in Sb_2_Te is ~5 at.%^26^. To understand why AIST favors this particular structural configuration, the formation energies can be obtained from density-functional theory. The corresponding calculations show that this structure, in which both Ag_Sb_ and In_Sb_ defects are located in the quintuple layers, is energetically favorable, as displayed in Fig. 4a. More specifically, the total electronic energy of M1 model with both Ag_Sb_ and In_Sb_ substituents inside the quintuple layer is −401.18 eV, which is 0.71 eV and 0.92 eV lower than those for the M2 (with one In_Sb_ substituent inside bilayer) and the M3 model (with one Ag_Sb_ substituent inside bilayer), respectively. We also find that Ag and In atoms always prefer to be located in the Sb sites of the quintuple layers in AIST as isolated dopants (see Supplementary Fig. 6**)**.

Further projected crystal orbital Hamilton population (pCOHP) bonding analysis^27^ in Fig. 4b confirms that the In–Te bonds in model M1 are slightly more stable, by ca. 0.8 eV, than the In–Sb bonds in M2, as reflected by the integrated pCOHP values of ‒12.8 and ‒13.6 eV. The latter (IpCOHP) values are the bonds’ contributions to the band-structure energy (equivalent to the sum of the Kohn‒Sham eigenvalues). In this very Fig. 4, spikes to the right denote bonding, and those to the left indicate antibonding levels^28^. While this is in line with the total energy of the system, the trend is not reproduced for Ag–Te and Ag–Sb interactions because the latter come out stronger by 0.5 eV, suggesting Ag cannot be stabilized through interactions of covalent character, which COHP analysis mirrors. To address this, additional Mulliken charge analysis was performed also using LOBSTER^29,30^. The results of this analysis are summarized in Table 1, where we observe a strong difference between the two dopants. While In carries very little charge (and a slightly negative one, so behaving like a mild anion), Ag behaves as a typical cation, with charges between +0.39 and +0.45. This comes as unexpected in Pauling’s empirical electronegativities (In: 1.78, Ag: 1.93)^31^ but is well reflected in Mulliken’s electronegativity scheme (In: 1.76, Ag: 1.47)^32,33^ based on ionization energies and electron affinities.

**Table 1:** Mulliken charge analysis of the In and Ag-substituents in models M1–M3 as generated by LOBSTER

In−Te (M3)			In−Sb (M2)			In−Te (M1)			Ag−Sb (M3)			Ag−Te (M2)			Ag−Te (M1)			
Atom		Charge	Atom		Charge	Atom		Charge	Atom		Charge	Atom		Charge	Atom			Charge
**2**	**In**	**−0.09**	**2**	**In**	**−0.19**	**2**	**In**	**0.00**	**1**	**Ag**	**0.39**	**1**	**Ag**	**0.45**	**1**		**Ag**	**0.45**
77	Te	−0.21	15	Sb	0.01	76	Te	−0.27	15	Sb	−0.13	77	Te	−0.31	76		Te	−0.27
78	Te	−0.10	16	Sb	0.01	80	Te	−0.13	16	Sb	−0.06	78	Te	−0.24	78		Te	−0.24
84	Te	−0.10	33	Sb	0.01	82	Te	−0.27	33	Sb	−0.13	84	Te	−0.24	82		Te	−0.27
89	Te	−0.20	34	Sb	0.01	86	Te	−0.13	34	Sb	−0.06	89	Te	−0.30	90		Te	−0.24
95	Te	−0.20	52	Sb	0.02	92	Te	−0.13	52	Sb	−0.06	95	Te	−0.30	94		Te	−0.27
96	Te	−0.11	69	Sb	0.01	94	Te	−0.27	69	Sb	−0.13	96	Te	−0.24	96		Te	−0.24
	**Σ**	**−0.92**		**Σ**	**0.07**		**Σ**	**−1.20**		**Σ**	**−0.57**		**Σ**	**−1.63**			**Σ**	**−1.53**

Charges are given for the substituent (first row, bold), the first coordination sphere of the substituent, as well as the sum of the first coordination sphere (last row, bold)

This fundamental result of differing bonding character (practically neutral In: mostly covalent, but cationic Ag: electrostatic) suggest the interaction between the silver substituent and its first coordination shell are stabilized by ionic contributions, which is fully supported by the charge analysis of its first coordination sphere. A similar trend is found for Mulliken charge analysis of the four models using either single In or Ag atom, as show in Supplementary Table 1. These results illustrate that In substituents are more stable within the Sb_2_Te_3_ layer due to covalent contributions visible in the COHP bonding analysis. Ag atoms, on the other hand, are stabilized through ionic interactions in the immediate surrounding of the dopant. Therefore, both substituents are more stable between Te layers, but for different reasons: indium through covalent interactions and silver through ionic interactions.

### Amorphous structure

After analyzing the atomic arrangement in AIST and its underlying bonding rationale, we now determine the precise roles of Ag and In in the AIST crystallization as mirrored from DFT-MD (molecular dynamics) simulations. We first analyze the change of amorphous structural features when incorporating Ag or In into a given Sb-Te matrix. To uncover the individual role of Ag and In, systems with isolated Ag, Ag_8_Sb_128_Te_48_ (ASI), as well as In dopants, In_8_Sb_128_Te_48_ (IST), are investigated. Structural characteristics of pure Sb_128_Te_48_ (ST) are presented as a reference. Their structures are obtained in two steps: first, the crystal structures, comprising of Sb bilayers and Sb_2_Te_3_ quintuple layers, where Sb is replaced by substituents, are melted at 3000 K for 50 ps; second, the high-temperature structures are relaxed at 600 K for 100 ps, where the last 60 ps are used for structural feature analysis.

**Fig. 5:**
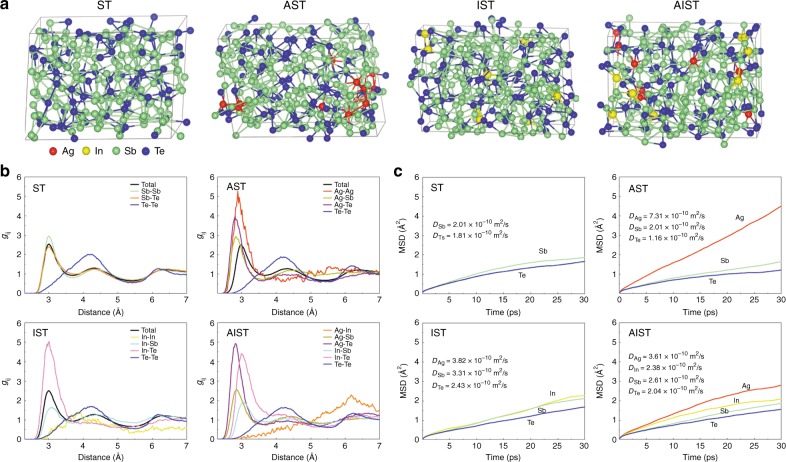
The structural and kinetic features of Sb_128_Te_48_, Ag_8_Sb_128_Te_48_, In_8_Sb_128_Te_48_, and Ag_8_In_8_Sb_128_Te_48_ systems at 600 K. **a** snapshots of the last frames of these systems relaxed at 600 K. **b** partial PDFs of these systems. **c** mean-square displacement (MSD) of these systems

Four atomic structures of the last frames at 600 K are shown in Fig. 5a. Upon melting, the ST model has lost all features of the layered crystal structure. Te, however, is almost exclusively surrounded by Sb, only very rarely by Te, which is confirmed by just 1.3% of homopolar Te−Te bonds found from the pair distribution function (PDF) in Fig. 5b. When adding ~4 at.% Ag to the ST model, the Ag atoms are located at the two sides of the amorphous AST structure, and none of them are found in the center. Moreover, Ag prefer to form clusters within amorphous AST (Fig. 5a), resulting in a strong first peak of Ag–Ag pairs near 3 Å (Fig. 5b). This configuration supports the phase segregation upon crystallization found by X-ray diffraction^34^. In contrast, indium in IST disperses homogenously in the matrix, and few In‒In bond are observed. This is supported by the very low first peak of an In−In pair near 4.0 Å in its PDF curve. Interestingly, the Ag clusters, observed in AST, completely disappear after adding In. In contrast to the homogenous distribution of indium in IST, In atoms are now located in the two ends of the amorphous AIST. As a result, the intensity of the first peak for Ag−Te pairs in AIST are even higher than in AST, whereas fewer Ag–Sb pairs are observed. The calculated PDF of the amorphous AIST is in good agreement with the experimental data obtained from electron diffraction (Supplementary Fig. 7), implying that the amorphous structure obtained from MD simulation accurately represents the experimentally observed amorphous phase. Thus, adding In prevents Ag atoms from the formation of Ag cluster. In other words, Ag/In atoms are highly localized around Te in amorphous AIST, with Sb filling up the remaining Te coordination sphere, as is observed in the crystalline phase. Note that the distribution of Te atoms is barely altered after incorporating Ag and In, which can be seen from quite similar PDF curves of Sb–Te/Te–Te pairs in ST and AIST. From the mean-square displacements (MSD) in Fig. 5c, we find that the diffusion coefficients (*D*_Ag_) of Ag (3.61 × 10^−10^ m^2^ s^−1^) and In (2.36 × 10^−10^ m^2^ s^−1^) atoms in AIST are half of that of the systems with isolated Ag (7.31 × 10^−10^ m^2^ s^−1^) or In (3.82 × 10^−10^ m^2^ s^−1^) substituents. This further proves that Ag and In atoms are mutually constrained in the AIST model. It is worthwhile to note that significantly reducing *D* would increase the activation energy *E*_*a*_ of amorphous phase (*D* ∝ 1/*E*_*a*_)^16^. Thus, both Ag and In play crucial roles in the stability of amorphous AIST, leading to its higher crystallization temperature compared to ST^35–37^. For all systems, the Te atoms possess the lowest *D*_Te_ and diffuse less compared to the other elements, which plays an important role in stabilizing the network structure. The structural and kinetic features of AIST clearly suggest that the two basic units in the crystalline phase, Ag/In and Sb-enriched areas, can keep their basic frameworks against thermal fluctuations at 600 K.

### Molecular-dynamics simulations of recrystallization

**Fig. 6:**
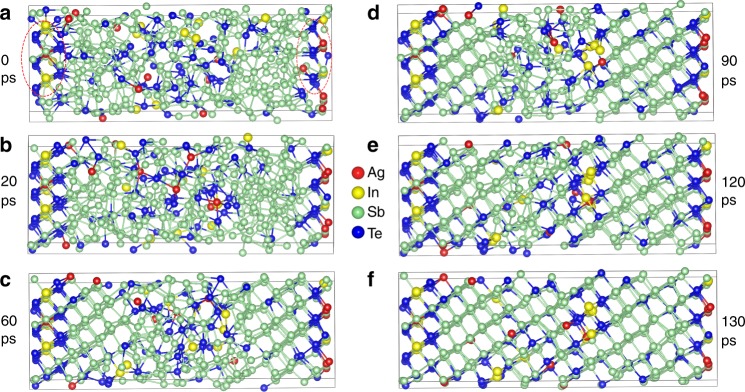
Structural evolutions of the crystallization process in AIST. **a**–**f** snapshots of the crystallization process of AIST at 0, 20, 60, 90, 120, and 130 ps (*T* = 600 K). Red circles highlight the introduced crystalline precursor made of Ag/In-Te cubes (45 atoms)

Following the above, determining how the Ag/In or Sb-enriched regions influence the crystallization is of interest. Since AIST is a growth-dominated PCM, here, we focus on the growth process of amorphous AIST. The structural evolution of amorphous AIST on annealing at 600 K were simulated using MD, as shown in Fig. 6a to f. To introduce the nucleus in this process, 45 atoms were fixed at the beginning, including 5 Ag, 5 In, 8 Sb, and 27 Te atoms. Once the crystalline precursors consisting of Ag/In-Te cubes (Fig. 6a) are formed, the precursors first grow along adjacent In atoms in the quintuple layers within the next 20 ps, which is therefore an incubation period for the crystallization. After this period, a clear amorphous-crystalline interface can be observed. The crystalline–amorphous rim grows toward the amorphous area, forming ordered bilayer structures, that is, aligning the Sb-centered octahedra along the *c* axes of the quintuple layer^12^ (Fig. 6b, c). This behavior shows the growth-dominated crystallization mechanism, a substantial difference from the nucleation-dominated mechanism found in GST. As presented in Fig. 6c, the right bilayers (4 layers/60 ps) grow faster than the left ones (3 layers/60 ps) with three Ag atoms. Compared to that, only ~30 ps are required to re-order the In-centered quintuple layer in the center (transition between Fig. 6d, e), while ~90 ps are necessary for the reconfiguration of three bilayers (Fig. 6a through d). This indicates a slight reduction in growth speed through Ag, whereas In accelerates crystallization. After 130 ps, an almost fully crystallized structure can be found, as shown in Fig. 6f. The vast majority of Ag and In atoms are located in the quintuple layer, quite closely resembling the observed crystalline structures in Fig. 2 and Supplementary Fig. 3. Subsequent short-order atom diffusion processes would occur to achieve the energetically favorable crystalline structure.

## Discussion

The ~3.9 at.% Ag, through forming unexpectedly ionic Ag–Te bonds, mutually constrain the random diffusion of the atoms in the Ag/In region. Both the ionic Ag–Te bonds and the mutually constrained effect are responsible for the higher crystallization temperature of AIST compared to ST. About 3.3 at.% In atoms accelerate the reconfiguration of quintuple layers, speeding up the crystallization process, which in turn results in the faster operation speed of AIST. To sum up, In and Ag in AIST interact to stabilize the amorphous phase, while In atoms speed up crystallization. After forming the Ag/In-enriched quintuple layers, the growth process is subsequently achieved via aligning the Sb-centered octahedra in Sb-enriched regions along the *c* axes of the quintuple layer, resulting in the growth-dominated crystallization behavior.

**Fig. 7:**
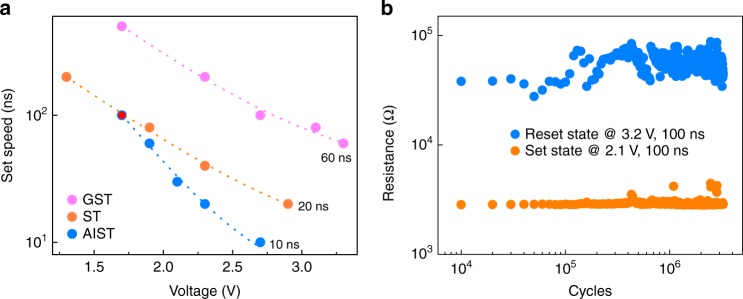
Device performance of optimized AIST (Ag_2.31_In_4.56_Sb_61.27_Te_31.86_) device. **a** set operation speed of GST, ST and optimized AIST-based devices. Optimized AIST presents the highest operation speed, 10 ns, comparable to DRAM. **b** endurance performance of optimized AIST device. The cells can be repeatedly operated over 2 million times

According to the aforementioned results, it is reasonable to lower the concentration of Ag in AIST to avoid possible phase segregation. In fact, AIST with 2.3 at.% Ag concentration already possesses sufficient stability against crystallization (at 173 °C). Device cells using the optimized AIST film show the transformation into a low-resistance state (crystallization state) in just 10 ns (Fig. 7a), comparable to DRAM. The speed is twice as fast as the ST-based cell, and six times faster than a GST-based one (see detailed information in Supplementary Fig. 8). The shortened switching process is achieved by acceleration of the crystallization process through In dopant engineering. Moreover, a reduced Ag content indeed prolongs the device lifetime of the cell, over three million cycles, as shown in Fig. 7b, one order of magnitude longer than an AIST-based cell (~10^5^ cycles).

We have now quantitatively determined the distribution of atomic-scale dopants in PCMs by advanced experimental and also theoretical techniques. Transferring the structure obtained into DFT calculations, the precise role of each element during crystallization was described and modelled. This accomplishment has been enabled by a detailed understanding of the crystallization behavior of Ag–In–Sb–Te alloys, in particular the role of small amounts of Ag and In. We thus hope that our work serves as a rational signpost for boosting phase-change memory through dopant engineering.

## Methods

### Film deposition and stoichiometry characterization

Six hundred nanometer thick AIST films were sputtered by alloy targets. The compositions of these films were identified by atom-probe tomography and arrived at Ag:In:Sb:Te = 5.13:3.32:65.60:25.93 in at.%.

### Cs-corrected TEM characterization

Amorphous AIST film was cut into TEM lamella using a dual-beam focused ion beam (FIB, FEI Helios Nanolab 600). The atomic-resolution STEM experiment was performed on a JEOL JEM-ARM300F microscopy equipped with a cold field-emission electron gun, dual-probe Cs-corrector, three detectors as well as an energy-dispersive X-ray (EDX) spectroscope. In situ like STEM investigations were performed to study the structural evolution of crystalline AIST. The investigation temperature ranges from room temperature to 350 °C. The sample was heated by TEM heating holder with a heating rate of 10 °C/min, which stopped at 200, 250, 300, and 350 °C, respectively. To get the atom-resolved mapping, this sample needed to be put into the EDX holder. Another sample was annealed by long-time annealing process, 300 °C for 30 min, and then investigated by Cs-corrected TEM to check the influence of annealing time on the atomic arrangement of crystalline AIST. The highest resolution of the Cs-corrected TEM, 0.63 Å, has been reported. The high-angle annular dark-field (HAADF) images were obtained at 80 kV or 300 kV, while EDX mappings were carried out at 80 kV. The size of EDX mapping were 1024 × 1024 pixels. The probe current was ~32 pA. The acquisition time was 30‒80 min.

To get the experimental structure information of the amorphous AIST, we extracted the pair distribution function (PDF) from the electron diffraction data of the amorphous AIST film. The electron diffraction pattern was obtained using JEOL JEM-ARM300F microscopy. The experimental data reduction techniques that used to obtain PDF can be found in refs. ^38,39^.

### Atom-probe tomography analysis

Six needled-sharped tips of crystalline AIST were prepared using the standard lift-out procedure by FIB, with a diameter of the apex less than 100 nm. These tips were mounted on a half-cut molybdenum TEM grid, which were then analyzed by JEOL JEM-2100F TEM in bright field mode as well as HAADF mode. The EDX mapping of these tips were also obtained. Subsequently, these tips were put into a 3 × 10^‒11^ mbar high vacuum in local electrode atom-probe (LEAP 5000 XR, Cameca Instruments). Applied a DC voltage of 2–6.5 kV and illuminated by 10 ps laser pulses in the atom-probe tomography, surfaces atoms of the needle-shaped sample were ionized, filed evaporated and subsequently projected onto a position-sensitive detector^24^. The *x*- and *y*-coordinates of the ions registered by the detector, as well as the calculated *z*-coordinate were used to reconstruct the 3D map. Moreover, these ions can be chemically identified based on time-of-flight mass spectrometry. Reconstruction and analysis of 3D maps were carried out using software IVAS 3.6.14.

### Device preparation and measurement

One hundred nanometer thick PCM film was deposited on the device with a bottom electrode diameter of 190 nm. Ten nanometer thick TiN and 300-nm-thick Al films are subsequently deposited on the PCM film, severed as the top electrode. The device performances were characterized by a parameter analyzer (Keithley 2400 C) and a pulse generator (Tektronix AWG5200B).

### Bond and charge computational details

Density-functional theory (DFT) calculations employed the ‘D3’ van der Waals correction^40^ on top of the PBE functional^41^. Plane-wave basis sets and the projector augmented wave (PAW) method^42^ were used as implemented in the Vienna Ab Initio Simulation Package (VASP)^43–45^. The energy cutoff for the plane-wave expansion was set to 500 eV with an electronic convergence criterion of 10^−7^ eV. Structural optimization was performed until residual forces fell below 5 × 10^−3^ eV Å^−1^ and reciprocal space was sampled on Γ-centered k-point grids with densities between 0.02 and 0.04 Å^−1^. Chemical-bonding analyses of plane-wave data, as well as Mulliken charge analysis, were performed using LOBSTER^27–29,46^.

### Molecular-dynamics simulations

Molecular-dynamics simulations were carried out based within the framework of density-functional theory (DFT-MD). The Kohn-Sham equations were solved using the VASP package and the Γ zone center only. The valence electron and core interactions were described using the projector augmented wave (PAW) method, where the valence electrons are 4*d*^10^5*s*^1^ for Ag, 4*d*^10^5*s*^2^5*p*^1^ for In, 5*s*^2^5*p*^3^ for Sb, and 5*s*^2^5*p*^4^ for Te. Electron exchange and correlation were treated as described above with a kinetic energy cutoff of 250 eV^40,41,47^. The convergence criterion was 2 × 10^‒5^ eV for electronic convergence and 0.2 eV/Å for force. Models were first melted at 3000 K for 50 ps with a time step *δt* = 2 fs, and then they were relaxed at 600 K for 100 ps. In the simulations, the Parrinello-Rahman (NpT) dynamics with Langevin thermostat was used. The recrystallization simulation model (384 atoms, including 16 Ag, 16 In, 256 Sb, and 96 Te atoms) with periodic boundary conditions was acquired as follows: 45 atoms, including 5 Ag, 5 In, 8 Sb, and 27 Te atoms, were fixed during melting and growing processes. The unfixed atoms were maintained at 3000 K for 15 ps to erase the information of crystalline state. The crystal-growth simulation for AIST was performed at 600 K. The total simulation time was 130 ps. The energy cutoff was 250 eV and the time step for the simulations was 3 fs.

### Mean-square displacement and Einstein relationship

The mean-square displacement is defined as

1 $${\mathrm{MSD}}_t = \frac{1}{N}\mathop {\sum }\limits_{i = 1}^N \left[ {x_i\left( {t + t_0} \right) - x_i\left( {t_0} \right)} \right]^2$$

where *N* is the total number of atoms, *x*_*i*_(*t*_0_) is the reference position of the *i*^th^ atom at time *t*_0_, *x*_*i*_(*t* *+* *t*_0_) is the position of the *i*^th^ atom at time (*t + t*_0_).

The diffusion coefficient *D* is determined according to the Einstein relationship

2 $${\mathrm{MSD}}_t = 2{\mathit{D}} \cdot {\mathit{t}}$$

where *t* is the time of atom movement.

## Supplementary information

Supplementary Information

## Data Availability

The data that support the findings of this study are available from the corresponding author upon reasonable request.
