# Supplementary material for: Direct atomic insight into the role of dopants in phase-change materials
Source: Nat Commun. 2019 Aug 6;10:3525. doi: 10.1038/s41467-019-11506-0 (PMC6684653; doi:10.1038/s41467-019-11506-0)
Supplement: Supplementary file 1 — Supplementary Information [file 41467_2019_11506_MOESM1_ESM.pdf]

Supplementary Information

Direct Atomic Insight into the Role of Dopants in Phase-Change Materials

Zhu et al.
*e-mail: minzhu@mail.sim.ac.cn

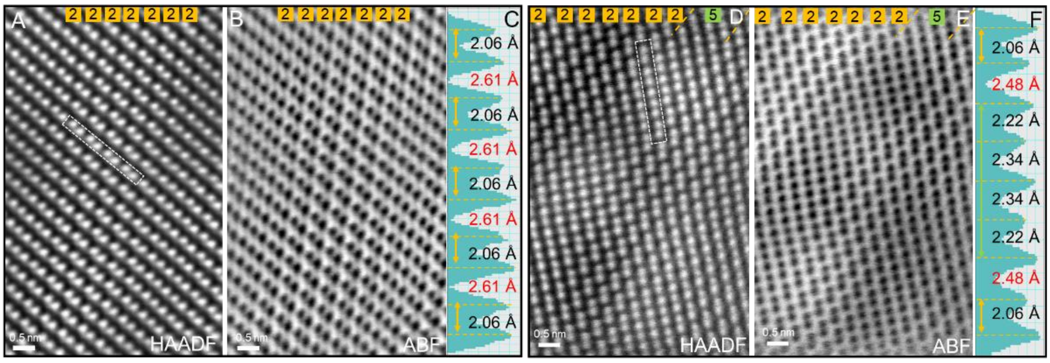

Supplementary Figure 1 Atomic arrangement of crystalline AIST phase-change materials, growing along the <001> direction. A: high-angle annular dark field-scanning transmission electron microscopy (HAADF-STEM) images of bilayer-like atomic stacking sequence in crystalline AIST film. B: corresponding annular bright field (ADF) of Figure A. C: intensity line profile of four-bilayer marked by dotted rectangle. D and E: HAADF-STEM and ADF-STEM images of quintuple layers (intermixing with bilayer), respectively. F: intensity line profile of bilayer-quintuple-layer-bilayer arrangement marked by dotted rectangle.

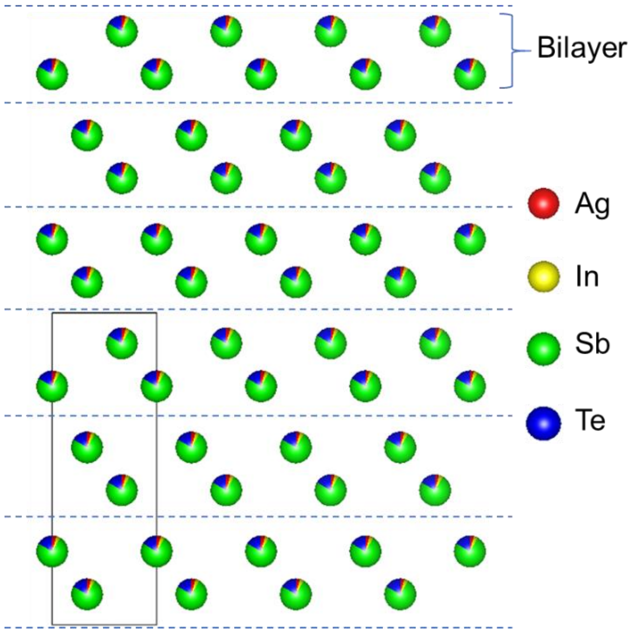

Supplementary Figure 2 Structure model of crystalline AIST projected along the <001> direction proposed by Matsunaga *et al*.[1] The structure has an *A7* structure with atoms of Ag, In, Sb or Te randomly occupying each of the atomic positions in space group $R\bar{3}m$. Bilayer-like stacking sequence can be seen in this structure. The dot lines mark a small 'gap' between the bilayers.

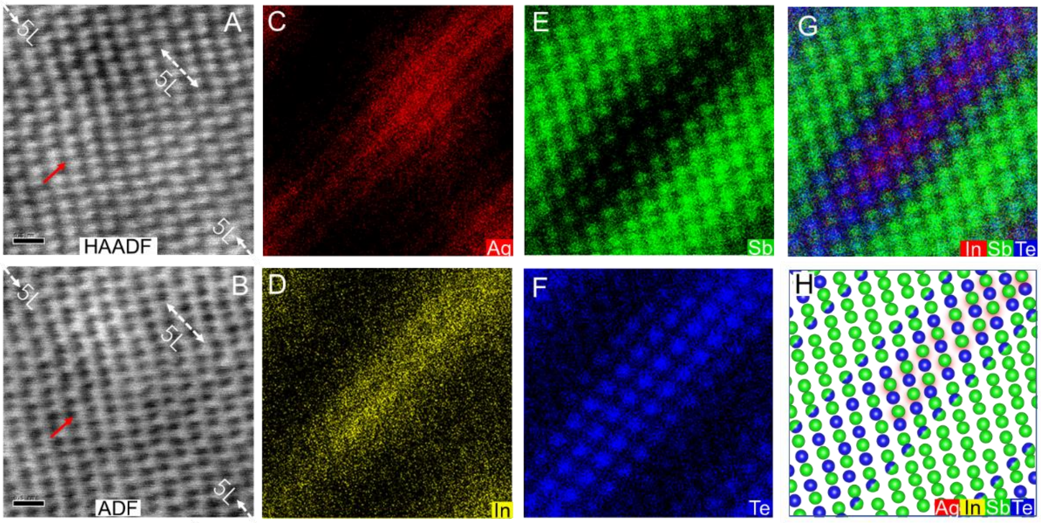

Supplementary Figure 3 Structural and chemical identifications of another AIST crystallite, stacked by bilayers and quintuple layers. A and B: HAADF-STEM and ADF-STEM images, respectively. The centered quintuple layer splits into 2-layer and 3-layer, marked by red arrow. C to G: EDX mappings for Ag, In, Sb, Te, and In/Sb/Te elements, respectively. H: corresponding atomic stacking model of crystalline AIST.

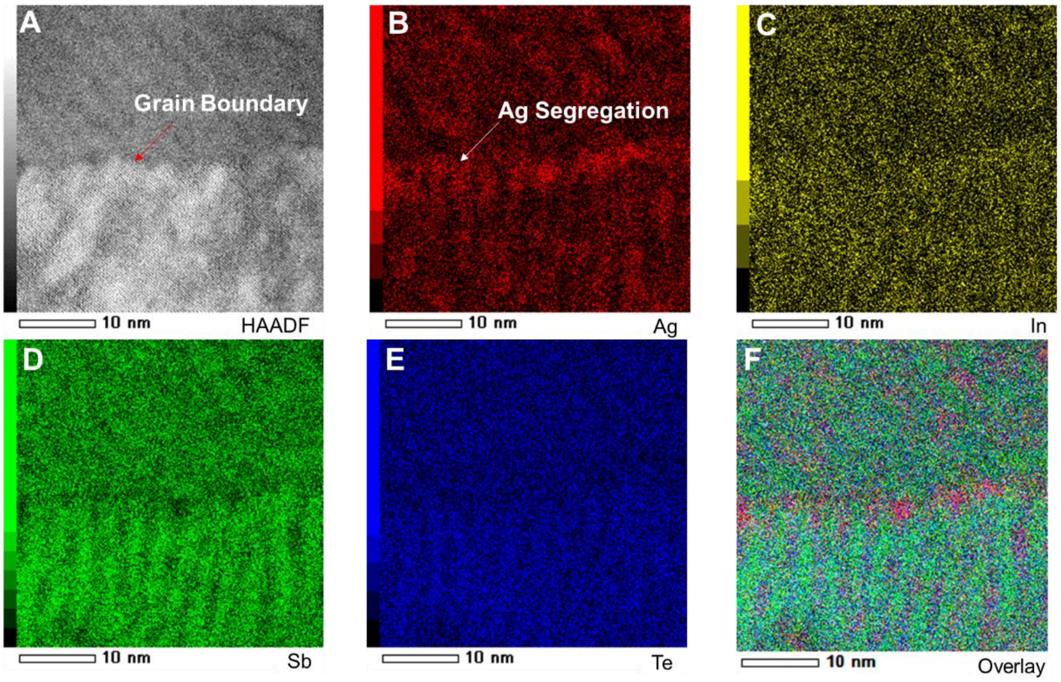

Supplementary Figure 4 Grain boundary of crystalline AIST annealed at 300 ^{o}C for 30 min. A: HAADF-STEM image of a grain boundary in crystalline AIST. B-F: EDX mappings for Ag, In, Sb, Te and overlaid Ag/In/Sb/Te elements. Ag atoms not only disperse in the crystalline lattices, but also aggregate in the grain boundary

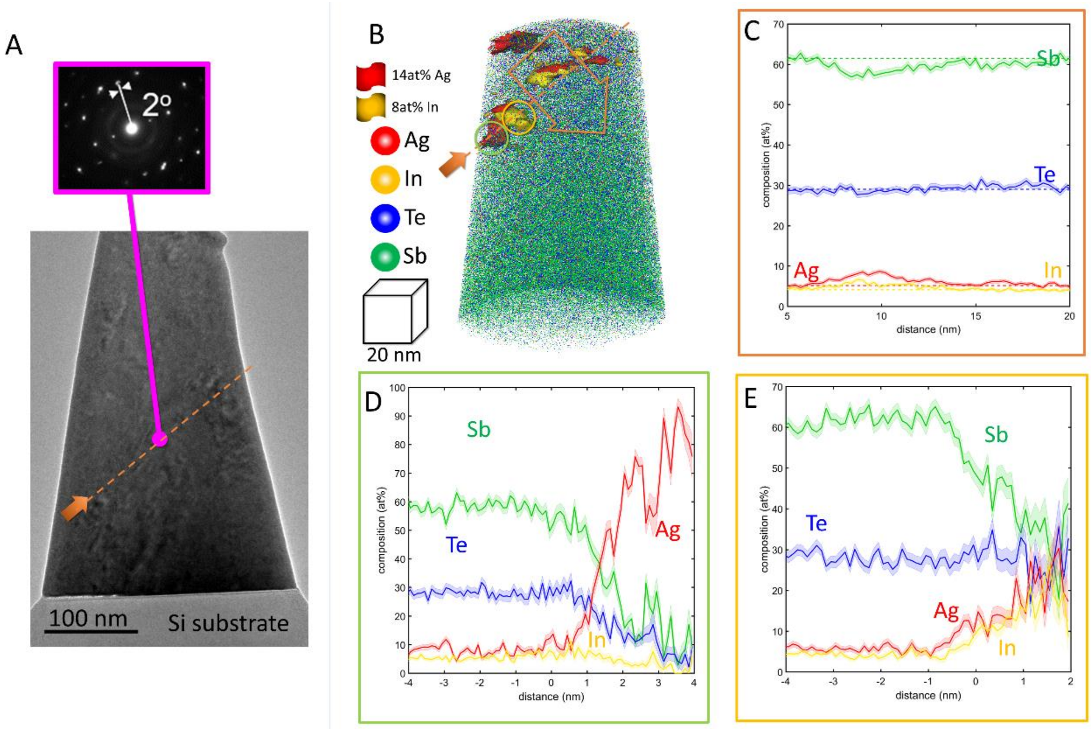

Supplementary Figure 5 Correlative APT-TEM investigation of low-angle grain boundary in crystalline AIST. A: HAADF-STEM image of a needle-shaped AIST tip. The inset is the diffraction pattern of the tip, implying the appearance of low-angle grain boundary (2°). The grain boundary is marked by a yellow arrow. B: reconstructed 3D maps of Ag, In, Sb and Te atoms of the tip obtained from APT. C: concentration profiles and ion count of the region of interest highlighted by the yellow cylinder. Proximity histogram concentration profiles of Ag, In, Sb and Te for D: 7.5 at.% Ag and E: 5.5 at.% In iso-concentration surfaces.

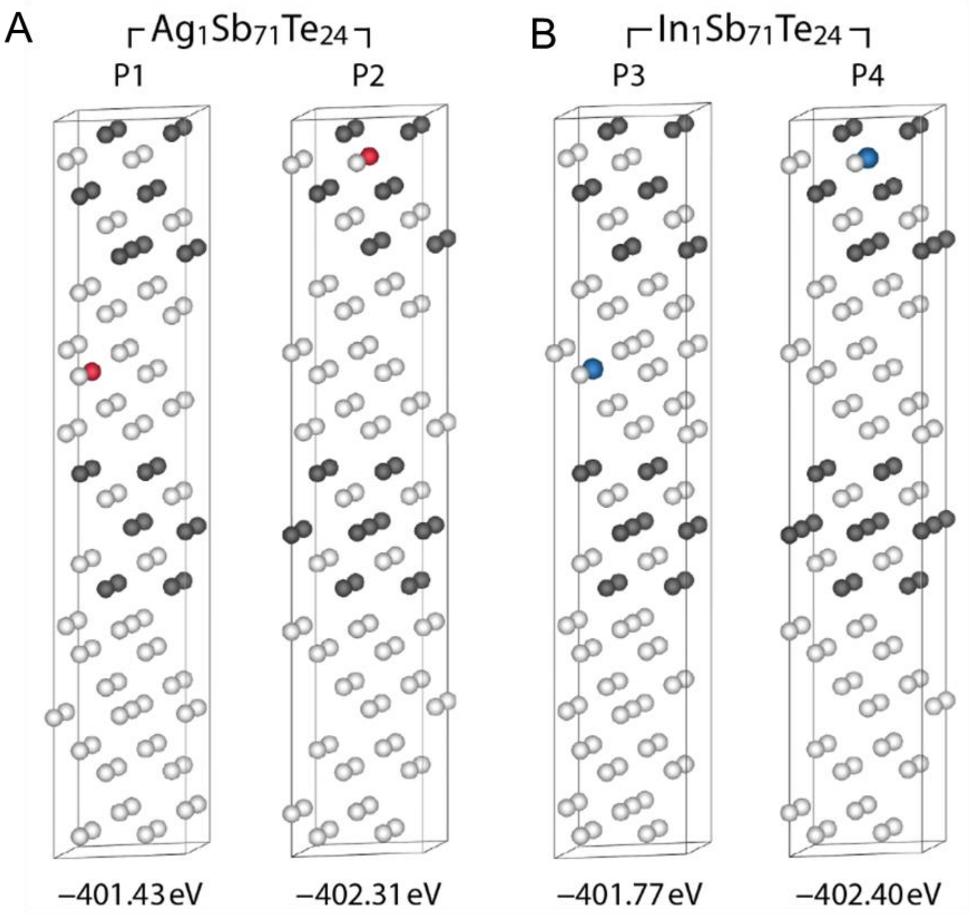

Supplementary Figure 6 Structural images of $Sb_{71}Te_{24}$ models with isolated Ag or In dopants (P1-4). The total energy always slightly lowers after the formation of Ag_{Sb} or In_{Sb} substitution defects in quintuple layer.

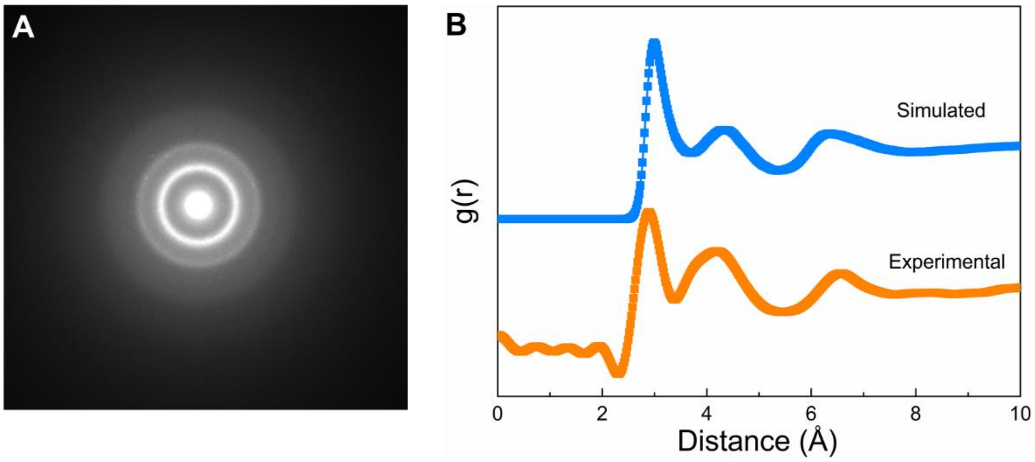

Supplementary Figure 7 Comparison of the experimental and DFT simulated structure information of the amorphous AIST. A: Electron diffraction pattern of the amorphous AIST obtained from TEM. B: Comparison of the experimental PDF with the data obtained from DFT simulation. The method for obtainning the PDF from electron diffraction pattern has been described in Reference 2 and 3.

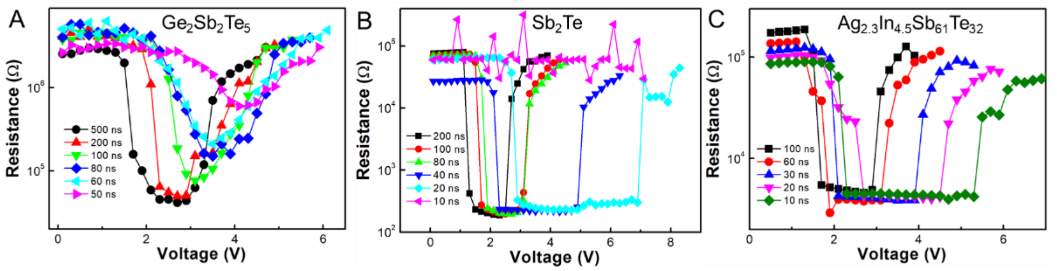

Supplementary Figure 8 Comparison of device performances of A: $Ge_2Sb_2Te_5$, B: Sb_2Te, and C: $Ag_{2.3}In_{4.5}Sb_{61}Te_{32}$-based cells. The diameter of the bottom electrode is ~190 nm. The GST-based device cannot be fully crystallized using a 50 ns pulse, while the ST-based device cannot be operated using a 10 ns pulse. This clearly shows that the fastest operation speed of GST and ST is ~60 ns and ~20 ns, respectively. Noticeably, the optimized AIST cell can achieve full crystallization using only a 10 ns pulse.

Supplementary Table 1. Mulliken charge analysis as generated by LOBSTER. Four models (P1–4) using either a single In or Ag atom were generated to exclude proximity effects. Silver behaves much more as a cation within the charge analysis, while indium accepts electron density. Therefore, Ag-dopants are likely stabilized by ionic contributions instead of covalent ones. Charges are given for the dopant (first row, bold), the first coordination sphere of the dopant, as well as the sum of the first coordination sphere (last row, bold).

Ag–Sb (P1)		Ag–Te (P2)		In–Sb (P3)		In–Te (P4)	
Atom	Charge	Atom	Charge	Atom	Charge	Atom	Charge
**1 Ag**	**0.40**	**1 Ag**	**0.45**	**1 In**	**−0.20**	**1 In**	**−0.08**
15 Sb	−0.15	77 Te	−0.31	15 Sb	0.00	77 Te	−0.21
16 Sb	−0.05	78 Te	−0.24	16 Sb	0.01	78 Te	−0.10
33 Sb	−0.15	84 Te	−0.24	33 Sb	0.00	84 Te	−0.10
34 Sb	−0.05	89 Te	−0.31	34 Sb	0.01	89 Te	−0.21
52 Sb	−0.05	95 Te	−0.31	52 Sb	0.01	95 Te	−0.21
69 Sb	−0.15	96 Te	−0.24	69 Sb	0.00	96 Te	−0.10
**Σ**	**−0.60**	**Σ**	**−1.65**	**Σ**	**0.03**	**Σ**	**−0.93**

Supplementary Reference:

[1] Matsunaga T., Umetani, Y., and Yamada, N. Structural study of a $Ag_{3.4}In_{3.7}Sb_{76.4}$Te16.5 quadruple compound utilized for phase-change optical disks. *Phy. Rev. B* **64**, 184116 (2001).

[2] Mitchell, D. R. G. and Petersen, T. C. RDFTools: a software tool for quantifying short-range ordering in amorphous materials. *Microsc. Res. Tech.*, 75, 153 (2012).

[3] Zhu, M., Xia M., Song, Z., Cheng, Y., Wu, L., Rao, F., Song, S., Wang, M., Lu, Y. and Feng, S. Understanding the crystallization behavior of the as-deposited Ti-Sb-Te alloys through real-time radial distribution functions. *Nanoscale*, 7, 9935 (2015).
